# Accumulation of oxysterols in the erythrocytes of COVID-19 patients as a biomarker for case severity

**DOI:** 10.1186/s12931-023-02515-1

**Published:** 2023-08-23

**Authors:** Alaa Khedr, Maan T. Khayat, Ahdab N. Khayyat, Hany Z. Asfour, Rahmah A. Alsilmi, Ahmed K. Kammoun

**Affiliations:** 1 Department of Pharmaceutical Chemistry, Faculty of Pharmacy, King Abdulaziz University, P.O. Box 80260, 21589 Jeddah, Saudi Arabia; 2 Department of Microbiology and Medical Parasitology, Faculty of Medicine, King Abdulaziz University, P.O. Box 80200, 21589 Jeddah, Saudi Arabia; 3 Department of Internal Medicine, Faculty of Medicine, King Abdulaziz University Hospital, King Abdulaziz University, P.O. Box 80200, 21589 Jeddah, Saudi Arabia

**Keywords:** COVID-19, Thrombosis, 7-Ketocholesterol, 4-Cholestenone, Acylcarnitines, Erythrocytes, Ion-trap-mass spectrometry, Oxysterols

## Abstract

**Background:**

Due to the high risk of COVID-19 patients developing thrombosis in the circulating blood, atherosclerosis, and myocardial infarction, it is necessary to study the lipidome of erythrocytes. Specifically, we examined the pathogenic oxysterols and acylcarnitines in the erythrocyte homogenate of COVID-19 patients. These molecules can damage cells and contribute to the development of these diseases.

**Methods:**

This study included 30 patients and 30 healthy volunteers. The erythrocyte homogenate extract was analyzed using linear ion trap mass spectrometry combined with high-performance liquid chromatography. The concentrations of oxysterols and acylcarnitines in erythrocyte homogenates of healthy individuals and COVID-19 patients were measured. Elevated levels of toxic biomarkers in red blood cells could initiate oxidative stress, leading to a process known as Eryptosis.

**Results:**

In COVID-19 patients, the levels of five oxysterols and six acylcarnitines in erythrocyte homogenates were significantly higher than those in healthy individuals, with a p-value of less than 0.05. The mean total concentration of oxysterols in the red blood cells of COVID-19 patients was 23.36 ± 13.47 μg/mL, while in healthy volunteers, the mean total concentration was 4.92 ± 1.61 μg/mL. The 7-ketocholesterol and 4-cholestenone levels were five and ten times higher, respectively, in COVID-19 patients than in healthy individuals. The concentration of acylcarnitines in the red blood cell homogenate of COVID-19 patients was 2 to 4 times higher than that of healthy volunteers on average. This finding suggests that these toxic biomarkers may cause the red blood cell death seen in COVID-19 patients.

**Conclusions:**

The abnormally high levels of oxysterols and acylcarnitines found in the erythrocytes of COVID-19 patients were associated with the severity of the cases, complications, and the substantial risk of thrombosis. The concentration of oxysterols in the erythrocyte homogenate could serve as a diagnostic biomarker for COVID-19 case severity.

**Graphical abstract:**

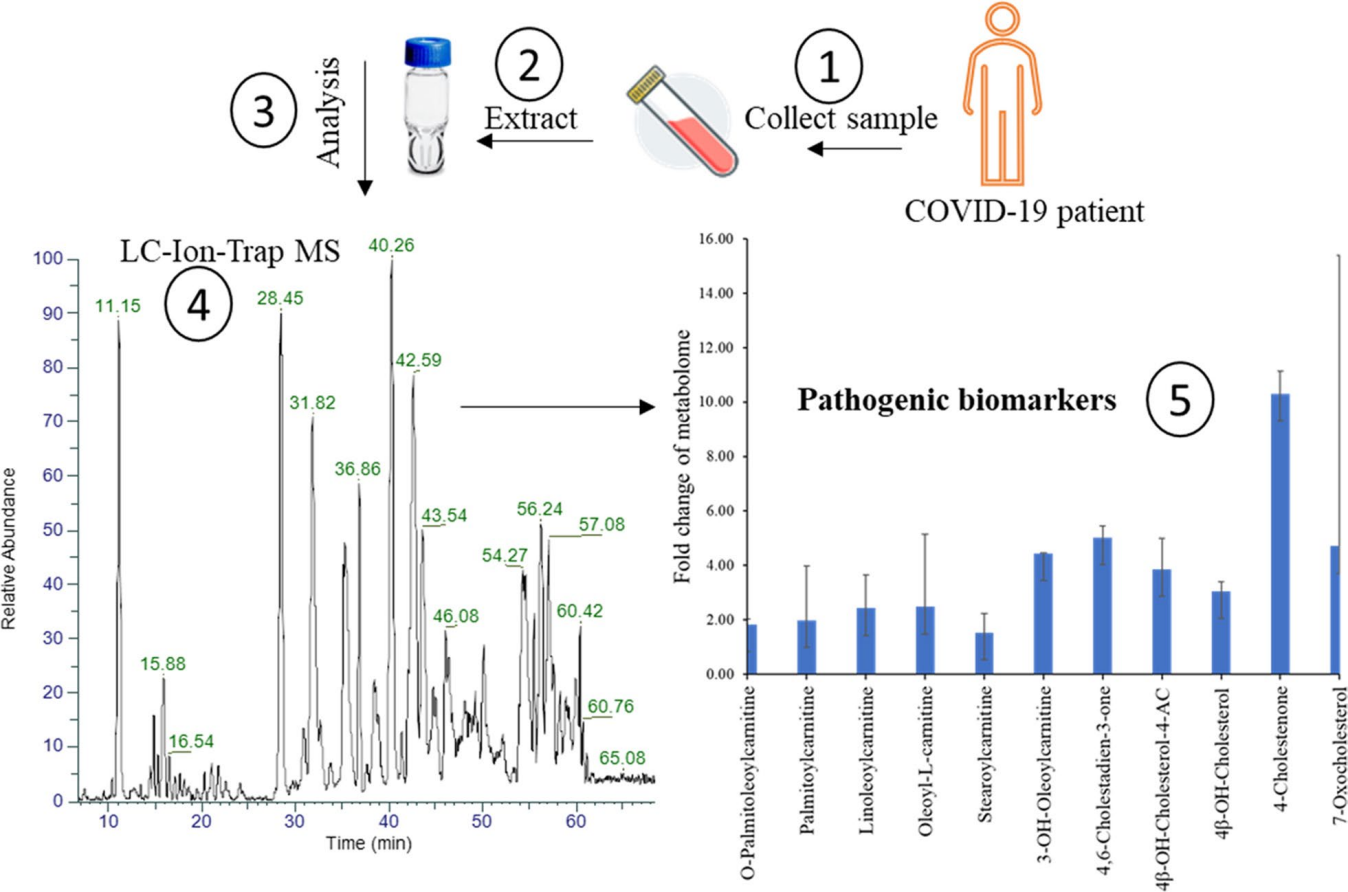

**Supplementary Information:**

The online version contains supplementary material available at 10.1186/s12931-023-02515-1.

## Introduction

The coronavirus pandemic started in the Chinese city of Wuhan, and the disease condition was defined as COVID-19 by the WHO on February 11, 2020. COVID-19 and its complications are well described by the World Health Organization [1, 2]. COVID-19 is caused by severe acute respiratory syndrome coronavirus 2 (SARS-CoV-2). The general clinical symptoms of this viral infection are fever, myalgia, general fatigue, and dry cough [3]. Some patients suffer more aggressive illnesses leading to pneumonia, breathing dysfunction [4], and cardiac injury, which might end with death [5]. COVID-19 can cause inflammation and damage to the mediastinal tissues between the lungs. This inflammation can lead to the formation of lesions, which are areas of abnormal tissue [6].

The severity of COVID-19 can vary widely. Severe cases can be life-threatening and lead to complications in multiple organs, including the lungs, heart, and kidneys. These complications are thought to be caused by a condition known as cytokine release syndrome [7]. COVID-19 patients are highly prone to blood hypercoagulation [8]. To date, the pathophysiology and mechanism of hypercoagulation are not understood [9]. However, many hypercoagulation mechanisms have been postulated and attributed to the impact of endothelial cell injury, the activation of the coagulation factor, inflammation, and the inactivation of fibrinolysis [10]. Lucijanic et al. found that a smaller proportion of COVID-19 patients present with thrombocytosis than with thrombocytopenia [8]. Alghareeb et al. enumerated several factors that can lead to the death of red blood cells, a process called eryptosis [11]. These factors include exposure to oxidative stress and changes in the composition of the membrane phospholipids due to lesions or some infections. Recent reports have shown that autopsies of COVID-19 patients revealed evidence of pulmonary endothelial viral inclusions, increased angiogenesis, apoptosis, and increased alveolus-microthrombi [12].

Oxysterols are the oxidation products of cholesterol. Oxysterols are important in the biological process of cholesterol homeostasis, apoptosis, and autophagy lipid metabolism [13]. The blood level of oxysterol may be considered a diagnostic biomarker for specific diseases or a predictor of the incidence of certain diseases, such as multiple sclerosis, osteoporosis, Alzheimer's disease, psychomotor diseases, and cancer [13, 14]. The accumulation of oxysterols as an end product of cholesterol results in chronic pathogenesis accompanied by cellular damage, as in neurodegenerative disease, congestive heart failure, and acute viral infections [15]. Some oxysterols exert potent oxidation damage to cells and acute inflammatory effects at low concentrations detected in the lesions of atherosclerosis, acute coronary syndrome, and neurodegenerative diseases [16]. The development of atherosclerosis can be attributed to oxidized lipids, such as oxidized low-density lipoprotein (ox-LDL) [17, 18]. High cholesterol levels in the blood can also contribute to the development of this condition [19].

7-Ketocholesterol is a harmful byproduct of cholesterol that is mainly produced through autooxidation [20]. It can cause oxidative stress in various diseases related to viral infections and some rare conditions. High levels of 7-ketocholesterol play a role in cell death because, at high concentrations, 7-ketocholesterol can cause oxidative stress, inflammation, and cell degeneration, which are all typical characteristics of these diseases [20]. 7-Ketocholesterol is primarily considered a substantial contributing cause of atherosclerosis via its detrimental effects on the macrophages that clear lipids from blood vessel walls [21]. 7-KCh exerts a toxic effect and induces cell damage [16]. Because of these different toxic activities, 7-KCh has been suggested to contribute to the pathogenesis of COVID-19 [22].

It has been postulated that the assay level of 7-KCh in plasma could be used as a prognostic biomarker for identifying the severity of COVID-19. This information could help identify patients at risk of worse clinical outcomes and optimize their medical care. Thus, it could decrease the number of patients needing mechanical ventilation [16]. In severe cases of COVID-19, it has been hypothesized that 7-KCh could be a biomarker for predicting disease severity and its potential complications [21]. Tang et al. reported that the lipidomic analysis of erythrocytes collected from heart failure patients revealed oxysterol accumulation [23]. Additionally, patients with high 7-KCh levels in plasma have shown substantially more dead giant cells that make high incidences of thrombosis [24]. It has been reported that both 7-ketocholesterol and 7β-cholesterol are moderately increased in the serum of COVID-19 patients using gas chromatography‒mass spectrometry as an assay method [25].

Samples intended to analyze cholesterol and oxysterol should be carefully handled since these compounds are oxidized by air within 30 min [26]. Acylcarnitine translocase exists in the extracellular space and is autoactivated to transport long-chain fatty acids into mitochondria for β-oxidation to be used by the cells as an energy source [27]. Abnormal expression of some acyl-Co enzyme-A leads to the accumulation of some acylcarnitines, which produces a toxic effect on the cell [27]. Important changes in acylcarnitine metabolism have been found in diseases such as dementia [28], heart failure [29], and coronary artery disease [30].

Barberis et al. 2020 demonstrated that COVID-19 patients had high concentrations of eighteen acylcarnitines in the plasma [31]. They showed that mitochondria secreted higher levels of acylcarnitines, characterized by long-chain fatty acids, which could exhibit a high-risk factor for lung injury with uncontrolled fatty acid oxidation. The accumulated lipids in the air fluid could increase the infection severity [32]. It has been described that viruses that infect the respiratory system, such as the flu virus, induce acylcarnitine accumulation, and a subsequent increase in acylcarnitine levels in the plasma of COVID-19 patients might be connected to this mechanism.

Herein, we investigated the correlation between COVID-19 and abnormal biogenic materials. The lipidomic profiles of cholesterol, some characteristic oxysterols, and acylcarnitines were studied to explain and justify the pathogenesis, respiratory dysfunction, and atherosclerosis. Because of the key role of acylcarnitines in cellular metabolism in different diseases, they could be expressive diagnostic or prognostic biomarkers. Therefore, we studied the lipidomics of acylcarnitines in COVID-19 patients because it can help enhance the evolution of disease diagnosis and treatment technology. The erythrocyte homogenate was used as the targeted sample for investigation in COVID-19 patients to monitor the oxidized cholesterol derivatives that might generate a multithrombotic particle and the possible formation of plaque [33, 34].

## Material and methods

### Study design and participants

Patients suffering from the general symptoms of COVID-19, including fever, chest pain with respiratory complaints, and general fatigue, were immediately subjected to polymerase chain reaction (PCR) testing. The nasopharyngeal swab sample was collected from the COVID-19 candidate and exposed to the PCR testing method because of its high specificity and sensitivity [35]. This study included only hospitalized patients who were clinically diagnosed and categorized as having moderate and severe COVID-19 in compliance with the international guidelines of COVID-19 case severity [36]. Accordingly, the selected patients were classified as either moderately or severely infected. Patients diagnosed as asymptomatic were excluded. The study exclusion criteria included pregnant women, volunteers receiving any hypolipidemic drugs, patients with diabetes, patients who received streptokinase injections, and acute cases under mechanical ventilation.

Each group involved 30 volunteers, with an equivalent number of males and females. The included volunteers were 40 to 75 years of age. They were admitted to the hospital and under medical supervision. Informed consent was read, understood, and signed by the patients. Healthy volunteers were also selected carefully and had recent medical investigation data at King Abdulaziz University Hospital, Jeddah, Saudi Arabia. The ethical committee approved the blood sample collection at King Abdulaziz University Hospital (reference number 408-20), and the National Committee of Biology and Medical Ethics registration number is "HA-02-J-008".

### Blood sample collection

A 5 mL blood sample was collected from the arm vein over sodium-EDTA, swirled, and immediately sent to the analysis lab. The blood samples were centrifuged at 3000 rpm for 15 min. The supernatant plasma was removed with a Pasteur pipette, transferred to a 7-mL brown-glass vial, and kept at -80 °C. The red blood cell fraction was mixed with 5 mL saline (0.9% sodium chloride in water, w/v), gently swirled, and centrifuged at 3000 rpm/10 min. The upper aqueous layer was removed and discarded. The remaining erythrocytes were then vortexed for 1 min and left in a sonication bath for 15 min to homogenize the ruptured erythrocytes. The samples of the homogenate of the red blood cells were extracted immediately or otherwise kept at −80 °C until analysis. The samples were stored in 7-mL brown glass vials, flushed with nitrogen gas, labeled, and tightly screw-capped before storage or analysis.

### Handling of blood samples

Due to the observed fragility of the red blood cells collected from infected patients, samples were collected and centrifuged with careful measures in place. Patients who participated in this study were classified clinically as moderate or severe COVID-19 infections [36]. The blood samples collected from COVID-19 patients were centrifuged at a slow speed of less than 3000 rpm. The COVID-19 samples that were separated at a faster centrifugal rate, > 3400 rpm/5 min, showed the fragility of red blood cells, as evidenced by the appearance of red hemoglobin in the supernatant plasma fluid. Nitrogen gas was used during the erythrocyte separation, extraction, and storage processes to avoid the formation of oxysterols due to air oxidation. COVID-19 patients on mechanical ventilation were excluded from the study due to the possibility of oxidizing biomaterials formed from exposure to oxygen in the inhaled air, which could lead to unreliable results.

### Standard materials

Standard lipids were obtained from Avanti Polar Lipids, Inc. (AL, USA), and the purity of each was more than 99.0%. The lipids included cholesterol, 4β-hydroxycholesterol-4-acetate, 4β-hydroxycholesterol, 7-ketocholesterol (7-KCh), 4-cholestenone (4-Chn), 4,6-cholestadien-3-one, O-palmitoleoylcarnitine (C16:1_CA), palmitoyl-L-carnitine (C16:0_CA), linoleoyl-L-carnitine (C18:2_CA), oleoyl-L-carnitine (C18:1_CA), stearoyl-L-carnitine (C18:0_CA), and 3-hydroxyoleoyl-carnitine (3OH-C18:1_CA). Three deuterated compounds, including oleoyl-L-carnitine-d9 (IS1), cholesterol-d6 (IS2), and 7-KCh-d7 (IS3), were purchased and used as internal standards.

### Instruments and conditions

A Thermo Scientific LTQ-XL linear ion trap mass spectrometer coupled with Accela autosampler and Accela pump (San Jose, CA, USA) was utilized. The ion source was the electrospray ionization (ESI) compartment. The system was controlled with Xcalibur® Thermo Fisher Scientific Inc., version 2.07 SP1. The spray voltage was 5.0 kV, sheath gas flow rate was 42 mL/min, auxiliary gas was 10 mL/min, capillary voltage was 60 V, and capillary temperature was 325 °C. The collision energy was 35 v. The Column was an Eclipse Plus C18 column of 3.5 μm, 4.6 × 100 mm (Agilent, Palo Alto, USA). The column oven was set at 40 ± 3 °C, and the tray temperature was 20 °C. The mobile system was composed of (A) water: methanol: ammonium hydroxide solution 25% (75: 25: 0.4, v/v), (B) methanol: chloroform: ammonium hydroxide solution 25% (95: 5: 0.4, v/v) and (C) methanol: chloroform: ammonium hydroxide solution 25% (75: 25: 0.4, v/v). The flow rate was 400 μL/min. The pump was programmed at 0–2 min to deliver 65% A, then decreased to 35% A at 9 min, decreased to 15% A at 30 min, decreased to 5% A at 40 min, and decreased to 1% A at 49–70 min.

A more detailed methodology is found in the Additional file 1.

## Characterized oxysterols and acylcarnitines

**Fig. 1:**
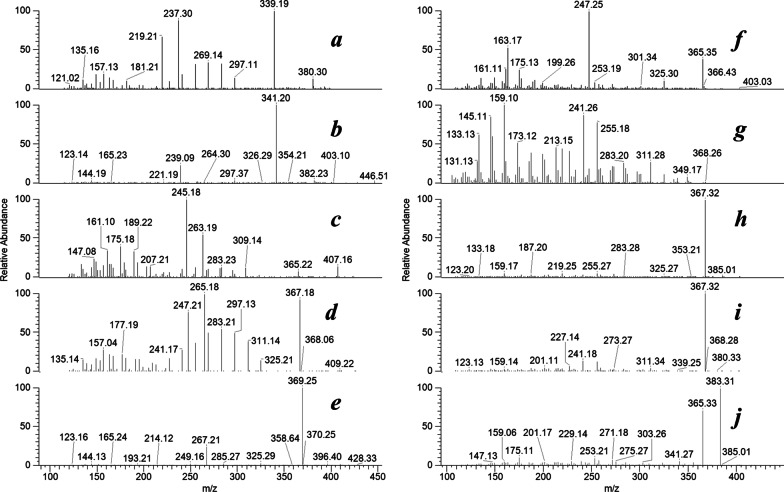
IT-MS^n^ spectra of the characterized lipidomes in the extract of erythrocytes obtained from COVID-19 patients. O-Palmitoleoyl-L-carnitine (**a**), palmitoyl-L-carnitine (**b**), linoleoyl-L-carnitine (**c**), oleoyl-L-carnitine (**d**), stearoyl-L-carnitine (**e**), 4,6-cholestadien-3-one (**f**), 4β-hydroxycholesterol 4-acetate (**g**), 4β-hydroxycholesterol (**h**), 4-cholestenone (**i**), and 7-ketocholesterol (j)

The IT-MS^2^ spectra of the detected precursor ions were investigated by NIST 2020 supported. The NIST database was updated to a release in 2022 using a retrievable NSIT-format online database of the Mass Bank of North America (MoNA) at https://mona.fiehnlab.ucdavis.edu. The IT-MS^n^ spectra of the confirmed lipidomes in the extract of erythrocytes obtained from COVID-19 patients were O-palmitoleoyl-L-carnitine (a), palmitoyl-L-carnitine (b), linoleoyl-L-carnitine (c), oleoyl-L-carnitine (d), stearoyl-L-carnitine (e), 4,6-cholestadien-3-one (f), 4β-hydroxycholesterol 4-acetate (g), 4β-hydroxycholesterol (h), 4-cholestenone (i), and 7-ketocholesterol (j), as shown in Fig. 1. The NIST similarity index of the characterized analytes was close to 95%. These identified biogenic materials were analyzed quantitatively in erythrocyte extracts collected from healthy individuals and COVID-19 patients.

More detailed information is in the Additional file 1, titled "Characterization of oxysterols and acylcarnitines".

### Solutions and calibration levels

A mixture of chloroform and methanol (2: 1, v/v) was used as a solvent matrix to prepare standard stock solutions and all other dilutions. One liter of this solvent was bubbled with nitrogen gas, 99.999%, for 1 min to expel any dissolved oxygen. The internal standard (IS) solution mixture was prepared in this solvent and contained 50 ng/μL of each oleoyl-L-carnitine-d9, cholesterol-d6, and 7-ketocholesterol-d7. The stock calibrant solution mixture containing 500 μg/μL each acylcarnitine, cholesterol, and oxysterol was prepared and kept at 4 °C in a glass vial sealed under nitrogen gas. A serial dilution mixture of this stock calibrant mixture was prepared to obtain a concentration range of 2 to 120 ng/μL for each analyte. A volume of 5 μL was injected for LC‒MS analysis. Seven calibration solution levels were prepared by mixing 25 μL of the internal standard solution mixture with 25 μL of the calibrant mixture. The final concentration of the calibrant spanned a range of 1 to 60 ng/μL with a constant concentration of each IS of 25 ng/μL.

### Calibration curve and assay method

Oleoyl-L-carnitine-d9 (+ *m/z* 435.4) was used as an internal standard for the targeted acylcarnitine assays, but cholesterol-d6 (+ *m/z* 375.3) was used as an internal standard for the cholesterol assays. Additionally, 7-ketocholesterol-d7 (+ *m/z* 408.3) was used as an internal standard for the oxysterol assays, including those for 7-KCh, 4-Chn, 4,6-cholestadien-3-one, 4β-hydroxycholesterol-4-acetate, and 4β-hydroxycholesterol. The calibration parameters are shown in Table 1. The calibration curves were constructed by plotting the MS-relative response of the analyte to the IS (Y-axis) and the concentration of the targeted analyte (X-axis). The squared regression coefficient was close to unity, and the response factor was close to 0.04. The ESI source was maintained and tuned daily at + *m/z* 524.33 to minimize ionization suppression due to ion source contamination. Samples that showed an analyte concentration outside the calibration range were diluted or concentrated by nitrogen gas to obtain an MS response within the valid range.

**Table 1:** Calibration parameters of the acylcarnitines and oxysterols in the homogenate of erythrocytes

t_R_, min	Name	Squared Regression coefficient, r^2^	Slope*	Intercept	Range, ng/μL	LOD, ng/μL
25.82	O-Palmitoleoyl-L-carnitine	0.9991	0.0425	0.0355	2.0–60	0.50
30.94	Palmitoyl-L-carnitine	0.9988	0.0399	0.0415	2.0–60	0.50
28.71	Linoleoyl-L-carnitine	0.9995	0.0517	0.0222	2.0–60	0.50
32.52	Oleoyl-L-carnitine	0.9999	0.0401	0.0066	2.0–60	0.50
37.64	Stearoyl-L-carnitine	0.9992	0.0452	0.0299	2.0–60	0.50
16.72	3-Hydroxyoleoylcarnitine	0.9987	0.0407	0.0425	2.0–60	0.50
32.55	Oleoyl-L-carnitine-d9, IS1	N/A	N/A	N/A	N/A	N/A
60.61	Cholesterol	0.9999	0.0417	0.0101	2.0–60	0.50
49.11	4,6-Cholestadien-3-one	0.9995	0.0398	0.0211	2.0–60	0.50
38.52	4β-Hydroxycholesterol-4-acetate	0.9997	0.0335	0.0198	2.0–60	0.50
38.82	4β-Hydroxycholesterol	0.9991	0.0392	0.0324	2.0–60	0.50
50.28	4-Cholestenone	0.9998	0.0378	0.0060	2.0–60	0.50
40.19	7-Ketocholesterol	0.9999	0.0414	0.0022	2.0–60	0.50
60.82	Cholesterol-d6, IS2	N/A	N/A	N/A	N/A	N/A
40.22	7-ketocholesterol-d7, IS3	N/A	N/A	N/A	N/A	N/A

^*^The regression line was drawn by plotting the concentration (X independent variable), ng/μL, versus the peak area ratio of the analyte to the internal standard (Y dependent variable)

### Sample preparation for the analysis

The glass vial containing the homogenate of erythrocytes was vortexed for 10 s. A volume of 100 μL was transferred to a screw-capped 15-mL glass test tube. It was mixed with 6 mL of the extraction solvent (chloroform: methanol, 2: 1, v/v) and 25 μL of the IS solution mixture and then left under a gentle stream of nitrogen gas for 30 s before capping. This mixture was vortexed for 2 min, sonicated for 15 min, vortexed for 30 s, and centrifuged at 5000 rpm for 15 min. The organic layer was separated by decantation to a 25-mL syringe fitted with a 0.22 μm PTFE filtration membrane. It was filtered into a 15-mL glass test tube and then dried with nitrogen gas over a water bath at 40 °C. The remaining residue was quantitatively transferred to a 1-mL total recovery vial using 200 μL of the same extraction solvent and gently bubbled with nitrogen gas to dryness. The residue was reconstituted in 50 μL chloroform and methanol; 2: 1, v/v. It was then capped under nitrogen gas and vortexed, and a volume of 5 μL was injected for LC-IT-MS analysis.

### Statistical analyses

The LCMS data were quantitatively processed using Thermo-Scientific Xcalibur, version 4.6.67.17. The output quantitative report and calibration data were saved as Microsoft Excel files. All univariate statistical parameters, including mean concentration, standard deviation, variance, and t-test, were calculated, and evaluated using Microsoft Excel. Samples that showed results out of the valid determination range were re-prepared at a higher or lower final expected concentration to be within the valid calibration range. The quantitative results of the targeted analytes were compiled separately for each subject and group. The individual data for each subject was collected to investigate the correlation between the severity of the case and the level of pathogenic markers or lipids. The statistically significant difference in lipid profile was calculated between healthy and COVID-19 patients.

## Results

### Extraction efficiency of erythrocytes

Special precautions have been taken because of the fragility of the red blood cells collected from COVID-19 patients. The blood samples centrifuged at 3200–3500 rpm showed a red supernatant plasma layer. The erythrocytes were separated at a slower centrifugal rate of 2800–3000 rpm to minimize the rupture of red blood cells. The applied extraction procedure considered not only the extraction yield and purity but also the nature and stability of the extracted biogenic material. The modified extraction process resulted in the highest lipid yield, which was protected from autooxidation due to using nitrogen gas during the entire procedure. More detailed information was added to the Additional file 1 "Optimization of the sample extraction".

Homogeneous erythrocytes collected from healthy volunteers were used as a matrix for preparing quality control (QC) samples. Four QC samples spiked with oleoyl-L-carnitine-d9, cholesterol-d6, and 7-KCh-d7 at high, median, low, and limit of quantification (LOQ) concentration levels (60, 20, 2, 0.2 ng/μL, respectively) were prepared and extracted. The analysis data of the QC samples, based on the MS-peak area, showed a mean percentage recovery of 95 ± 3.7% with a % error of not more than 4.0% for the three deuterated ISs. The IT-MS chromatograms were examined for any expected oxidation products of the spiked cholesterol-d6 due to bad sample handling. This procedure showed no oxidation products derived from the spiked cholesterol-d6. The coextracted unidentified biogenic materials were omitted by removing the unwanted + *m/z* ions and subtracting the LCMS base peak value from the extracted ion chromatogram.

### Quantitative results of oxysterols and acylcarnitines

**Fig. 2:**
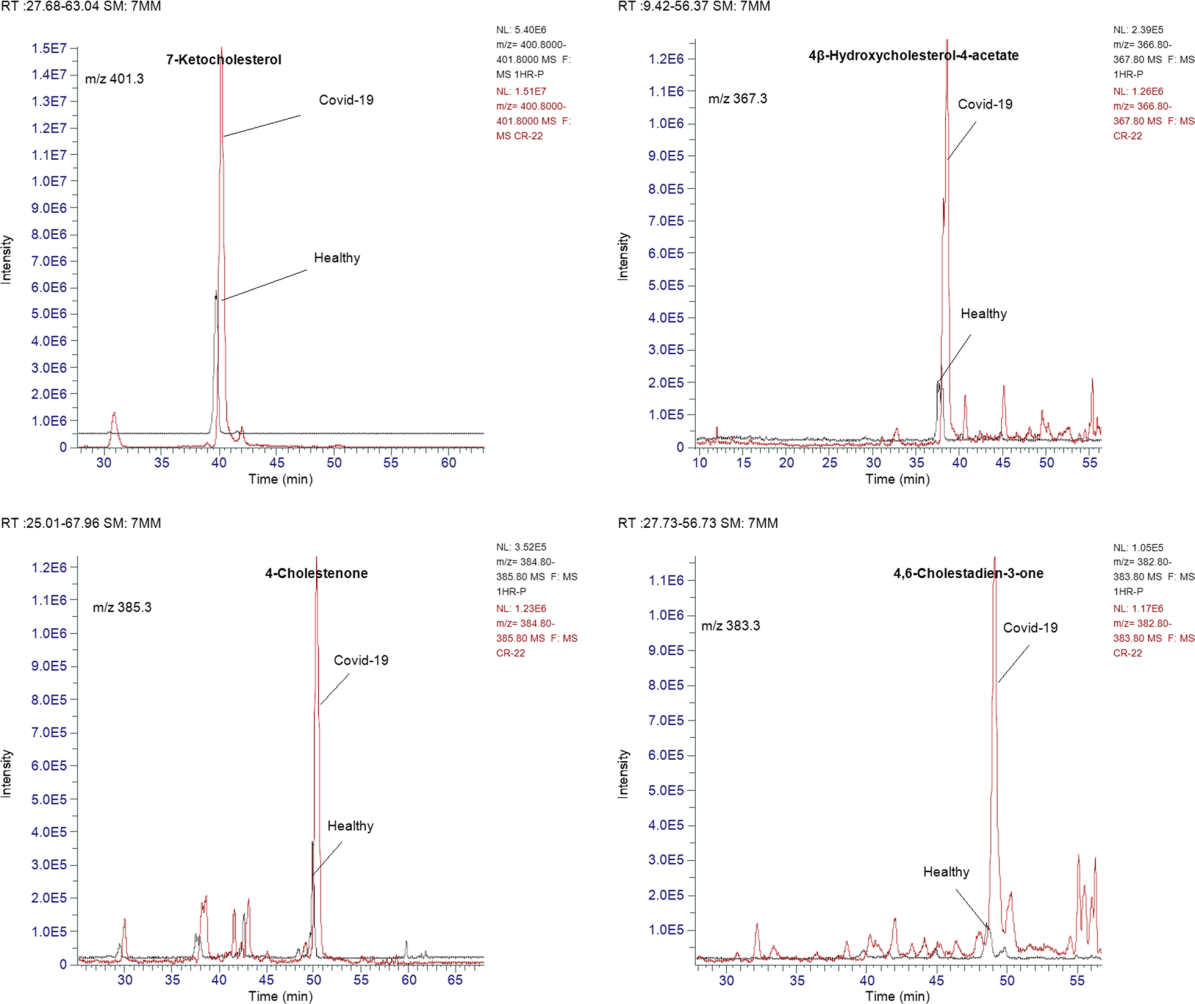
Representative overlayed-MS extracted ion chromatograms of oxysterols in healthy and COVID-19 patients' erythrocytes

**Fig. 3:**
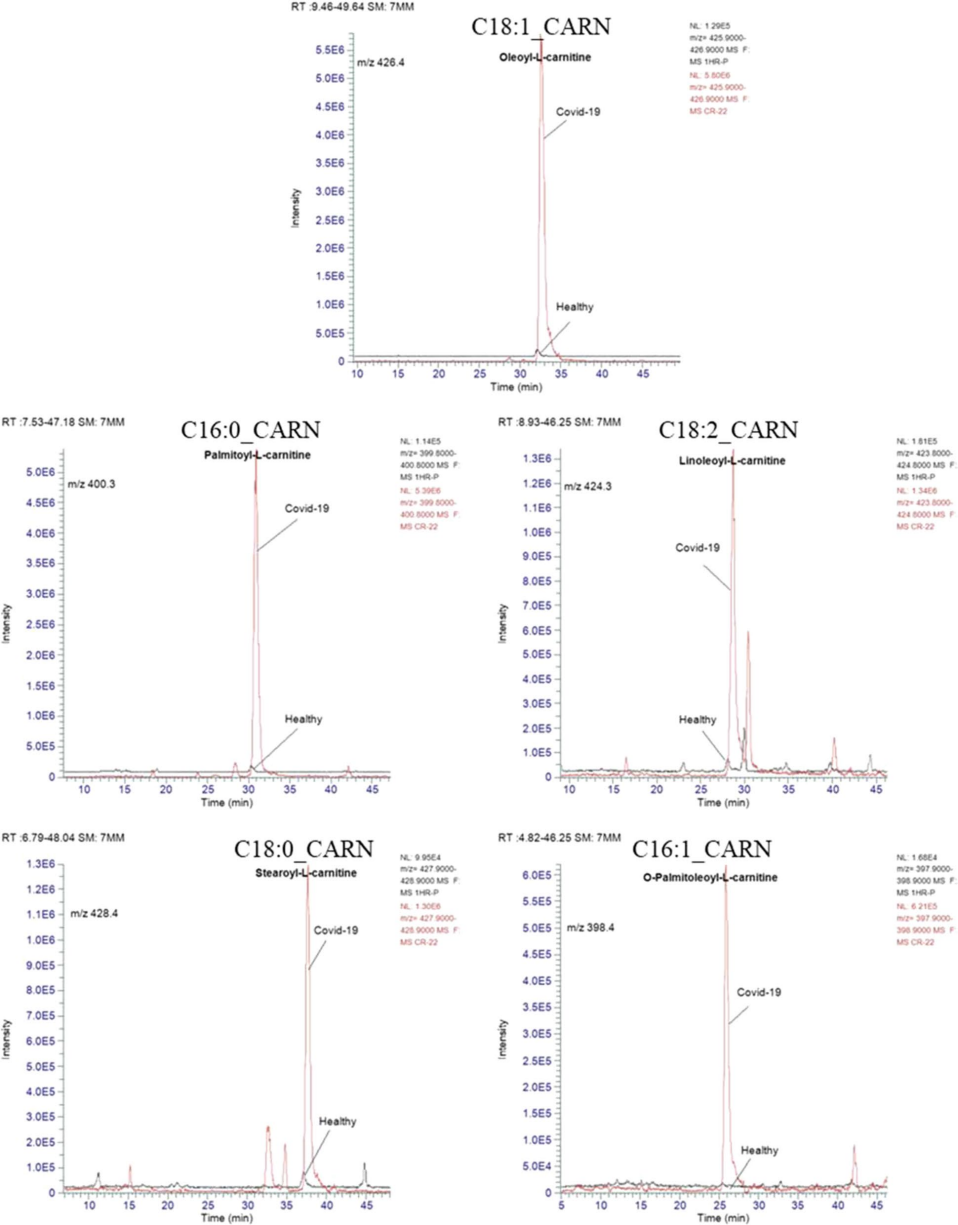
Representative overlayed-MS extracted ion chromatograms of acylcarnitines in healthy and COVID-19 patients' erythrocytes

The concentrations of the identified major acylcarnitines and oxysterols were calculated from the corresponding calibration curve (Table 1). The extracted ion chromatogram of each analyte was first identified, assigned, and integrated and then included within the list of peaks to be processed to calculate the concentration using Thermo-Xcalibur software (version 4.5.474.0, January 2022). The concentration of each analyte was calculated based on the relative peak area of the targeted compound to the assigned IS. Table 2 shows the mean concentrations of acylcarnitines, cholesterol, and oxysterols in erythrocytes of healthy volunteers and COVID-19 patients. Student's t-test was calculated, and the significant change in the values was computed at *p* values of 0.05, n = 30 for each group. Data revealed that the levels of acylcarnitines, cholesterol, and oxysterols were significantly increased in COVID-19 patients. The extracted ion chromatogram of all targeted lipidomes was relatively more intense in COVID-19 patients, matching the healthy volunteers (Figs. 2 and 3).

**Table 2:** The concentration of acylcarnitines, cholesterol, and oxysterols in erythrocytes of healthy volunteers and hospitalized COVID-19 cases. *p-*Values calculated at probability ± 0.05, n = 30 of each group

t_R_, min	Name	Exact mass	Precursor ion, + *m/z*	Healthy, concentration μg/mL ± SD		COVID-19, concentration, μg/mL ± SD		*p*-value	**
25.82	O-Palmitoleoyl-L-carnitine	397.32	398.32	0.19	± 0.22	0.35	± 0.20	0.01	↑
30.94	Palmitoyl-L-carnitine	399.33	400.33	1.96	± 0.69	3.88	± 2.01	0.00	↑
28.71	Linoleoyl-L-carnitine	423.33	424.34	0.91	± 0.44	2.20	± 1.24	0.00	↑
32.52	Oleoyl-L-carnitine	425.35	426.36	2.00	± 0.65	4.96	± 2.67	0.00	↑
37.64	Stearoyl-L-carnitine	427.36	428.38	1.07	± 0.36	1.64	± 0.69	0.00	↑
16.72	3-Hydroxyoleoylcarnitine	441.35	442.36	0.01	± 0.00	0.06	± 0.03	0.00	↑
32.55	Oleoyl-L-carnitine-d9, IS1	434.35	435.37	–		–		–	–
60.61	Cholesterol	386.35	369.37	0.06	± 0.01	0.19	± 0.09	0.00	↑
49.11	4,6-Cholestadien-3-one	382.32	383.34	0.19	± 0.05	0.95	± 0.43	0.00	↑
38.52	4β-Hydroxycholesterol-4-acetate	444.36	367.38	0.47	± 0.16	1.83	± 1.13	0.00	↑
38.82	4β-Hydroxycholesterol	402.35	385.37	0.17	± 0.13	0.52	± 0.37	0.00	↑
50.28	4-Cholestenone	384.34	385.36	0.15	± 0.13	1.50	± 0.85	0.00	↑
40.19	7-Ketocholesterol	400.33	401.33	3.94	± 1.14	18.56	± 10.69	0.00	↑
60.82	Cholesterol-d6, IS2	392.35	375.35	–		–		–	–
40.22	7-Ketocholesterol-d7, IS3	407.33	408.34	–		–		–	–

*Mean *m/*z value obtained with zooming-dependent fragmentation mode

**Statistical significance, ↑ significant increase

**Fig. 4:**
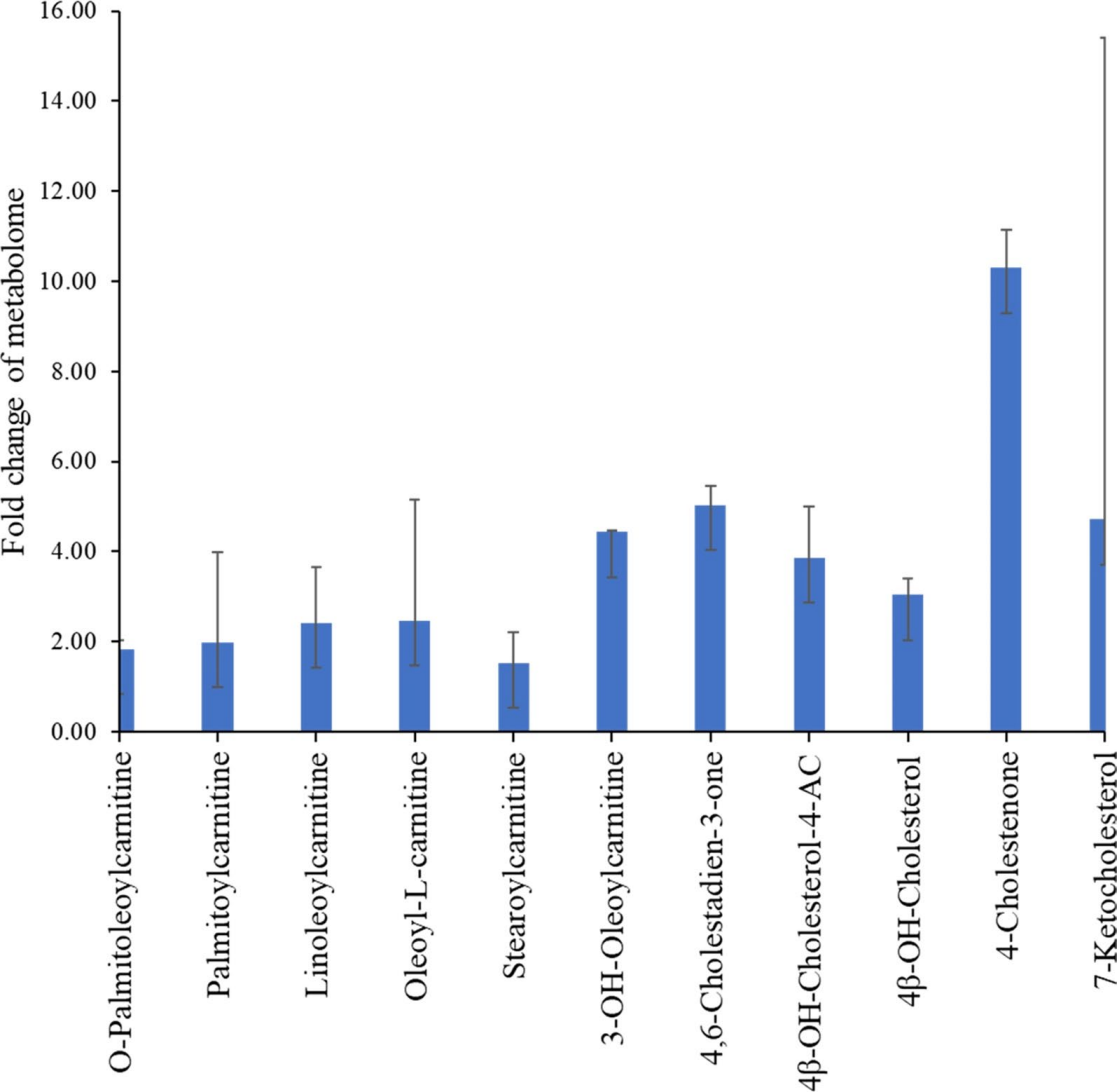
The fold change of oxysterol and acylcarnitine levels in the erythrocyte homogenate of COVID-19 patients compared to healthy volunteers

The erythrocyte homogenate collected from the 30 patients showed three ketonic and two hydroxylated cholesterol derivatives. The significantly increased levels of 7-ketocholesterol (also known as 7-oxocholesterol), 18.56 ± 10.69 μg/mL, and 4-cholestenone, 1.50 ± 0.85 μg/mL, were the highest levels and the most pathogenic biomarkers found in the erythrocyte homogenate of COVID-19 patients. Figure 4 displays the fold change of the detected oxysterols. The average concentrations of 4-cholestenone and 7-ketocholesterol increased tenfold and fivefold, respectively, compared to healthy individuals. Additionally, the significantly increased levels of oleoyl-L-carnitine, 4.96 ± 2.67 μg/mL, and palmitoyl-L-carnitine, 3.88 ± 2.01 μg/mL, were the highest levels among the six acylcarnitines found in COVID-19 erythrocytes.

Moreover, the analysis of erythrocyte homogenate in COVID-19 patients showed a significant increase in six acylcarnitines, as shown in Table 2. The concentration of the quantified acylcarnitines showed a 2- to fourfold increase, matching the data obtained from the healthy volunteers (Fig. 4).

**Fig. 5:**
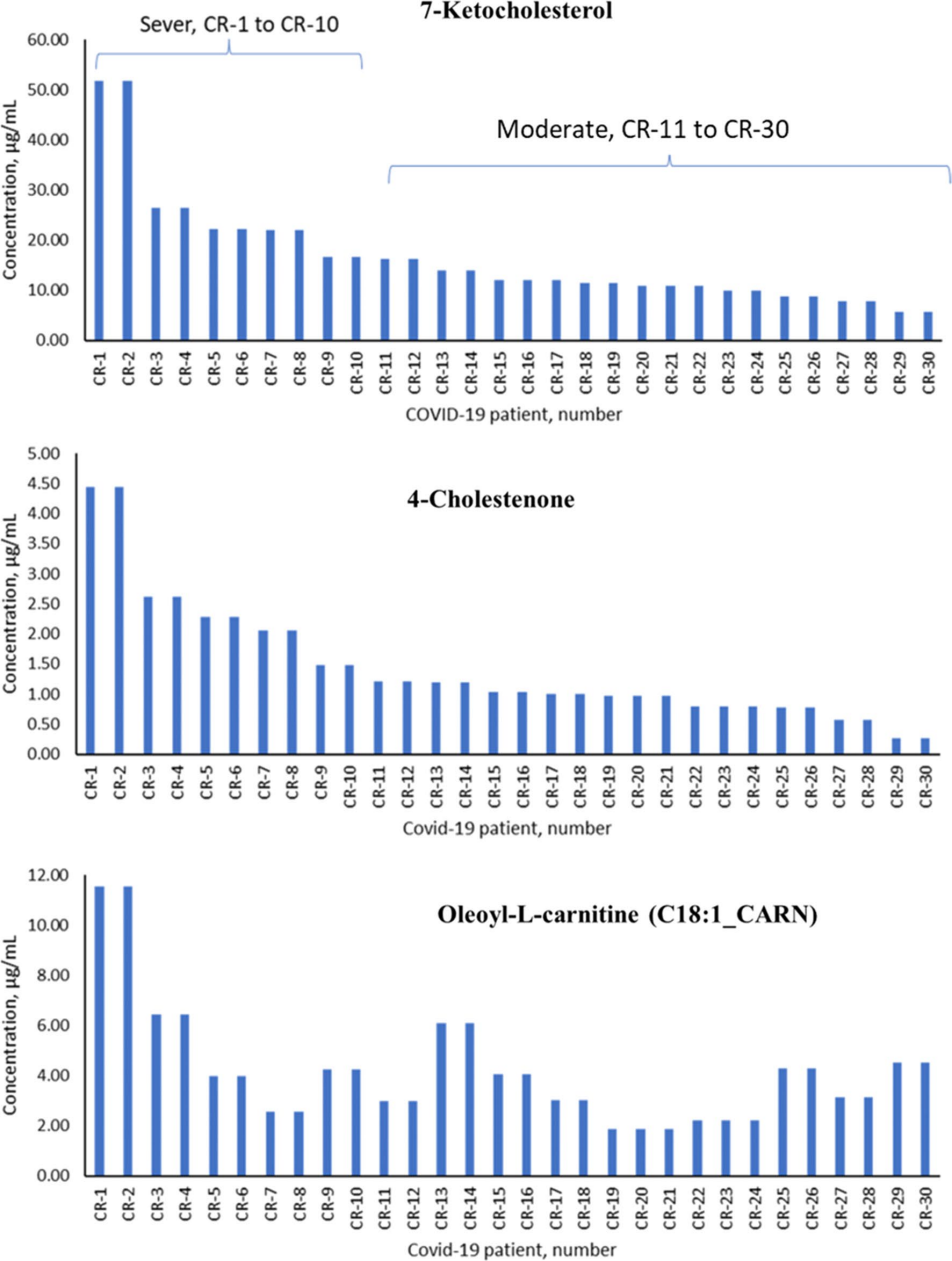
The severity of COVID-19 cases in relation to the levels of 7-ketocholesterol, 4-cholestenone, and oleoyl-L-carnitine in the erythrocyte homogenate

Figure 5 shows the median level of 7-ketocholesterol, 4-cholestenone, and oleoyl-L-carnitine in the erythrocytes' homogenate of COVID-19 patients grouped according to case severity. The level of oleoyl-L-carnitine showed no characteristic pattern in correlation with the case severity. However, the concentration of 7-ketocholesterol, and 4-cholestenone were relatively higher in severe cases compared with the patients classified as moderate.

## Discussion

The observed fragility of the RBCs could be attributed to the dysregulated lipid content of the cellular membrane. Samples that showed extra red pigmentation were re-extracted by a slower centrifugation rate of 2800 rpm for 15 min. Such samples were obtained from severe COVID-19 patients. Several extraction trials were conducted to obtain an extract with the highest yield and purity and lowest red pigmentation. The modified Bligh and Dyer extraction method was applied, which considered the extraction yield and purity and the nature and stability of the extracted biogenic material. Additionally, the oxidized lipids are the main reason for thrombosis formation in the lung and cardiovascular system, as previously reviewed. To guarantee that these oxylipids were released due to the viral infection and not air oxidation, it was necessary to use nitrogen gas in all sample handling procedures.

In this work, blood samples were collected from thirty patients clinically diagnosed with COVID-19 of moderate to severe stage and 30 healthy control individuals. The erythrocyte-homogenate samples were extracted and analyzed with ion trap-mass spectrometry. The IT-MS results confirmed the abnormal elevation of five oxidation products derived from cholesterol and six acylcarnitines (Fig. 1). A modified LC‒MS analytical procedure was applied to quantitatively estimate the pathogenic lipid metabolites. The extracted ion chromatogram showed relatively intense MS peaks corresponding to the precursor positive ions of oxysterols and acylcarnitines in erythrocytes collected from COVID-19 patients (Figs. 2 and 3).

The severity of COVID-19 has been reported to be associated with cell death, inflammation, N-glycosylation, and thrombosis, which are considered critical COVID-19 biomarkers [37]. The characterized analytes were quantitatively determined since these metabolites have been reported as erythrocytic pathogenic biomarkers released due to their harmful impact on biological cells [10, 21, 25, 38]. The summary of the results we obtained is consistent with the report published by Reva et al. [22]. The virus causing COVID-19 has been reported to target human red blood cells for replication and cause cell damage.

The applied methodology showed a valid calibration parameter of the assayed targeted analytes (Table 1). The quantitation results showed a significant increase in oxidized cholesterol and acylcarnitines (Table 2). 7-Ketocholesterol and 4-cholestenone were the oxysterols most highly associated with two hydroxylated cholesterol metabolites. 4-Cholestenone and 7-ketocholesterol were increased in the erythrocytes of COVID-19 patients by ten- and fivefold, respectively, compared with the levels found in healthy subjects.

Additionally, the significantly increased levels of oleoyl-L-carnitine, 4.96 ± 2.67 μg/mL, and palmitoyl-L-carnitine, 3.88 ± 2.01 μg/mL, were the highest levels among the six acylcarnitines found in COVID-19 erythrocytes. COVID-19 patients were organized according to the severity of the case based on the clinical diagnosis and the occurrence of complications. According to the WHO guidelines, the first ten cases were considered critical, and the rest were deemed moderate [36]. The concentrations of 7-KCh, 4-Ch, and C18:1_CARN were plotted versus patient numbers, as shown in Fig. 5. This figure shows that the more severe the COVID-19 case is, the higher the concentrations of accumulated oxysterol and C18:1_CARN. The concentration ranges of 7-ketocholesterol and 4-cholestenone in the erythrocytes of COVID-19 patients defined as severe were 52.0 to 16.6 and 4.5 to 1.5 μg/mL, respectively. However, the moderate COVID-19 patients showed concentration ranges of 16.2 to 5.7 and 1.4 to 0.3 μg/mL for 7-KCh and 4-Ch, respectively. The lowest concentrations of both 7-KCh and 4-Ch in the COVID-19 patients were higher than the mean values obtained from healthy volunteers, as shown in Table 2. The level of 7-KCh in the erythrocytes of moderate and severe cases was increased by 2.0–4.5- and 4.5–12.5-fold, respectively (Fig. 5).

Analysis of 7-KCh in erythrocytes was preferred over serum due to its relatively higher concentration, which facilitates measurement and early detection in mild cases of COVID-19. In this study, the mean amount of 7-KCh in the erythrocytes of healthy volunteers was 3.9 μg/mL. However, it has been reported that the serum concentration of 7-KCh was 20 ng/mL and increased in COVID-19 patients [21, 25]. Marcello et al. found that the serum level of 7-KCh was increased 2–3.5- and 2–fivefold in moderate and severe COVID-19 cases, respectively [25]. Based on these data, it could be concluded that 7-KCh was specifically significantly increased in both erythrocyte homogenate and serum.

4-Cholestenone (4-cholesten-3-one) is one of the intermediate oxidation products of cholesterol. It has been reported that 4-Chn is highly mobile in erythrocyte membranes, affecting cholesterol flip-flop and efflux. It has been reported that cholestenone formation may cause long-term functional defects in red blood cells [39]. Chandra et al. 2022 claimed that cholestenone is a unique oxidation product of cholesterol that exists extra and intracellularly in patients suffering from Mycobacterium tuberculosis infection [40]. This study confirmed that cholestenone, 4-cholestenone, accumulated in the erythrocyte membrane. Cholestenone could be detected at concentrations below 0.08 μg/mL in the plasma of COVID-19 patients classified as severe. The literature review showed no data identifying or quantifying 4-cholestenone in the blood of COVID-19 patients. Moreover, the LC‒MS analysis data confirmed that COVID-19 patients exhibited an accumulation of three ketonic cholesterols in the erythrocyte membrane, including 7-ketocholesterol, 4-cholestenone, and 4,6-cholestadien-3-one. This higher level of oxysterol found in the erythrocyte homogenate of COVID-19 patients could not only serve as a diagnostic biomarker for case severity, but it could also be considered an indicator of the aggregation of dead cells and subsequent high risk of developing multiple sclerosis in the respiratory system or circulating blood [16, 21].

Additionally, the severe COVID-19 cases exhibited a higher level of C18:1_CARN. COVID-19 patients showed a significantly higher concentration of oxysterol, which indicates oxidative stress and, subsequently, the risk of apoptosis [16, 21]. Acylcarnitine accumulation has been reported to have a toxic effect on biological cells [27]. Significant changes in acylcarnitine metabolism have been observed in diseases such as dementia [28], heart failure [29], and coronary artery disease [30]. Patients with moderate to severe cases of COVID-19 clinically suffer difficulty breathing, dementia, and heart problems. All these symptoms could be attributed to the accumulation of acylcarnitines.

The results suggest that the total concentrations of oxysterols alone or with acylcarnitines in erythrocytes can be a diagnostic biomarker for COVID-19. The high levels of these metabolic products may explain the high incidence of thrombosis and cellular damage in COVID-19 patients. These results support our hypothesis that COVID-19 patients are at increased risk of thrombosis, cellular damage, and oxygen deprivation.

## Limitations

We carefully adhered to the inclusion and exclusion criteria for COVID-19 patients to prevent sample bias. We also avoided ascertainment bias by classifying the selected patients into moderate and severe cases according to WHO guidelines [36]. These steps helped to ensure that the sample of patients used in the study was representative of the population of interest and that the diagnoses of COVID-19 were made consistently.

A precise analysis methodology was applied to ensure the validity of the data. Certain experimental precautions have been taken to account for the fragility of the erythrocytes' cell membrane and the possibility of air oxidation of the extracted lipids to get more reliable data with no confounding results. The accuracy and precision of such lipidomic studies are limited by the correct selection of the analytical technique and instrumentation that give explicit results. Mass spectrometry is the preferred technique for lipidomic studies. However, the accuracy of the instrumental response and, subsequently, the data accuracy can be affected by the complexity of the sample matrix [41]. In this study, ion trap mass spectrometry was preferred and applied because of its superiority in peak confirmation before quantification. This technique also enables the discovery of any relevant unusual compounds resulting from metabolic dysfunction.

## Conclusions

COVID-19 patients were found to have significantly higher levels of 7-ketocholesterol, 4-cholestenone, and six acylcarnitines in their erythrocyte homogenate. These lipidomes can harm biological cells, particularly red blood cells, and contribute to thrombosis formation. Based on the data collected and previous studies, it can be concluded that COVID-19 patients are at a high risk of developing thrombosis in the bloodstream, atherosclerosis, myocardial infarction, and pulmonary failure due to dysregulation of the lipid composition in erythrocytes. Furthermore, 7-ketocholesterol and 4-cholestenone could potentially serve as markers for the severity of COVID-19. The erythrocytes of severe COVID-19 patients had a concentration range of 52.0 to 16.6 μg/mL for 7-ketocholesterol (7-KCh) and 4.5 to 1.5 μg/mL for 4-cholestenone (4-Ch). In contrast, moderate COVID-19 patients had a concentration range of 16.2 to 5.7 μg/mL for 7-KCh and 1.4 to 0.3 μg/mL for 4-Ch. The minimum concentrations of both 7-KCh and 4-Ch in COVID-19 patients exceeded the mean values observed in healthy volunteers. The level of 7-KCh in erythrocytes increased by a factor of 2.0–4.5 in moderate cases and 4.5–12.5 in severe cases.

### Supplementary Information

**Additional file 1.** This file contains more detailed information about; instruments and conditions, characterization of oxysterols and acylcarnitines, and optimization of the sample extraction.

## Data Availability

The data that support the findings of this study are available from the corresponding author, A Khedr, upon reasonable request.

## Abbreviations

4-Chn: 4-Cholestenone
7-kCh: 7-Ketocholesterol
COVID-19: Coronavirus disease of 2019
ESI: Electrospray ionization
HPLC: High performance-liquid chromatography
IT-MS: Ion trap mass spectrometry
