# Supplementary material for: Accumulation of oxysterols in the erythrocytes of COVID-19 patients as a biomarker for case severity
Source: Respir Res. 2023 Aug 23;24:206. doi: 10.1186/s12931-023-02515-1 (PMC10464166; doi:10.1186/s12931-023-02515-1)
Supplement: Supplementary file 1 — Additional file 1. This file contains more detailed information about; instruments and conditions, characterization of oxysterols and acylcarnitines, and optimization of the sample extraction. [file 12931_2023_2515_MOESM1_ESM.docx]

**Additional file 1**

**Instruments and conditions**

Thermo Scientific LTQ-XL linear ion trap mass spectrometer coupled with Accela autosampler and Accela pump (San Jose, CA, USA). The ion source; is the electrospray ionization (ESI) compartment. The system was controlled with Xcalibur® Thermo Fisher Scientific Inc, version 2.07 SP1. Spray voltage, 5.0 kv, sheath gas flow rate, 42 mL/min, auxiliary gas, 10 mL/min, capillary voltage, 60v, capillary temperature, 325 °C. The collision energy was 35 v. Column, Eclipse Plus C18, 3.5 μm, 4.6 x 100 mm (Agilent, Palo Alto, USA), column oven, 40±3 °C. Tray temperature, 20 °C. The mobile system was composed of (A) water: methanol: ammonium hydroxide solution 25% (75: 25: 0.4, v/v), (B) methanol: chloroform: ammonium hydroxide solution 25% (95: 5: 0.4, v/v) and (C) methanol: chloroform: ammonium hydroxide solution 25% (75: 25: 0.4, v/v). The flow rate was 400 μL/min. The pump was programmed at 0 – 2 min to deliver 65%A, decreased to 35%A at 9 min, decreased to 15%A at 30 min, decreased to 5%A at 40, and decreased to 1%A at 49 – 70 min.

The ion trap-mass spectrometer (IT-MS) detector was programmed to monitor ions by applying positive scan mode, 100 - 1200 *m/z*, and dependent auto-fragmentation mode. MS^n^ spectra generation for ions exceeds the mass count of 1000. Data were saved as raw files for further investigation by Processing Setup, Thermo Xcalibur 4.5.474.0, 1/14/2022., and FreeStyle™ 1.8 SP2, Modern Data Visualization Software, Version 1.8.63.0, Build Date: Friday, July 30, 2021, Copyright © 2021 Thermo Fisher Scientific Inc. The NIST Mass Spectral Search Program, Version 2.4, build Mar 25, 2020. The NIST database was enriched by the online MassBank of North America (MoNA) and is metadata-centric (<https://mona.fiehnlab.ucdavis.edu>).

**Characterization of oxysterols and acylcarnitines**

The mean IT-MS^2^ spectra of the oxysterols and acylcarnitines were retrieved after a dependent IT-MS scan subjected to a collision energy of 35v and re-analyzed by assigning the peak of interest at the corresponding retention time with the zooming option. The IT-MS^2^ spectra of the detected precursor ions were investigated by NIST 2020 supported. The NIST database was updated to a release of 2022 using a retrievable NSIT-format online database of the Mass Bank of North America (MoNA) at [https://mona.fiehnlab.ucdavis.edu](https://mona.fiehnlab.ucdavis.edu/). The identified precursor ions showed a delta-mass difference of ±0.002 *m/z* applying targeted-zooming fragmentation mode.

The ionization efficiency and, subsequently, the detectability of cholesterol, oxysterols, and acylcarnitines at the ESI-MS was improved by investigating the effect of pH of the mobile system. The mobile phase containing ammonium hydroxide (0.4%, w/v) showed intense precursor ions of cholesterol, oxysterol, and acylcarnitine, while formic acid (0.1%, w/v) showed a very low ESI-MS response or little peaks of the targeted analytes. . The pH variation of the mobile phase does not affect the peak retention time; however, an intense and precise peak response was observed in the presence of ammonia solution.

The IT-MS^n^ spectra of the confirmed lipidomes in the extract of erythrocytes obtained from Covid-19 patients were; O-palmitoleoyl-L-carnitine (a), palmitoyl-L-carnitine (b), linoleoyl-L-carnitine (c), oleoyl-L-carnitine (d), stearoyl-L-carnitine (e), 4,6-cholestadien-3-one (f), 4β-hydroxycholesterol 4-acetate (g), 4β-hydroxycholesterol (h), 4-cholestenone (i), 7-ketocholesterol (j) as shown in Fig. 1. The NIST similarity index of the characterized analytes was close to 95%. These identified biogenic materials were analyzed quantitatively in the erythrocyte extract collected from healthy and COVID-19 patients.

**Optimization of the sample extraction**

Several extraction trials have been reported to recover the highest percentage of lipids and cholesterols from the erythrocytes [1, 2]. All reported extraction trials have been conducted to obtain an extract with the highest yield, purity, and low red pigmentation. This work applied a modified Bligh and Dyer [3] extraction method. The applied extraction procedure considered not only the extraction yield and purity but also the nature and stability of the extracted biogenic material. Auto-oxidation of the biomolecules was avoided throughout the sample preparation procedure. All extraction procedures were performed under nitrogen gas to avoid misleading results. Samples that showed extra-red pigmentation were re-extracted by a slower centrifugation rate of 2500 rpm for 15 min. Such samples were obtained from a severe COVID-19 case.

**References**

1. Rose HG, Oklander M: **Improved procedure for the extraction of lipids from human erythrocytes.** *J Lipid Res* 1965, **6:**428-431.

2. Tang HY, Wang CH, Ho HY, Wu PT, Hung CL, Huang CY, Wu PR, Yeh YH, Cheng ML: **Lipidomics reveals accumulation of the oxidized cholesterol in erythrocytes of heart failure patients.** *Redox Biol* 2018, **14:**499-508.

3. Bligh EG, Dyer WJ: **A rapid method of total lipid extraction and purification.** *Can J Biochem Physiol* 1959, **37:**911-917.
